# HPV vaccines: a controversial issue?

**DOI:** 10.1590/1414-431X20155060

**Published:** 2016-04-08

**Authors:** A.F. Nicol, C.V. Andrade, F.B. Russomano, L.L.S. Rodrigues, N.S. Oliveira, D.W. Provance

**Affiliations:** 1 Laboratório Interdisciplinar de Pesquisas Médicas, Instituto Oswaldo Cruz, Fiocruz, Rio de Janeiro, RJ, Brasil; 2 Instituto Nacional de Saúde da Mulher, da Criança e do Adolescente Fernandes Figueira, Fiocruz, Rio de Janeiro, RJ, Brasil; 3 Instituto de Saúde Coletiva, Universidade Federal do Oeste do Pará, Santarém, PA, Brasil; 4 Hospital Universitário Antonio Pedro, Universidade Federal Fluminense, Niterói, RJ, Brasil; 5 Centro de Desenvolvimento Tecnológico em Saúde, Fiocruz, Rio de Janeiro, RJ, Brasil

**Keywords:** Human papillomavirus, Vaccination, Adverse events

## Abstract

Controversy still exists over whether the benefits of the available HPV vaccines outweigh the risks and this has suppressed uptake of the HPV vaccines in comparison to other vaccines. Concerns about HPV vaccine safety have led some physicians, healthcare officials and parents to withhold the recommended vaccination from the target population. The most common reason for not administering the prophylactic HPV vaccines are concerns over adverse effects. The aim of this review is the assessment of peer-reviewed scientific data related to measurable outcomes from the use of HPV vaccines throughout the world with focused attention on the potential adverse effects. We found that the majority of studies continue to suggest a positive risk-benefit from vaccination against HPV, with minimal documented adverse effects, which is consistent with other vaccines. However, much of the published scientific data regarding the safety of HPV vaccines appears to originate from within the financially competitive HPV vaccine market. We advocate a more independent monitoring system for vaccine immunogenicity and adverse effects to address potential conflicts of interest with regular systematic literature reviews by qualified individuals to vigilantly assess and communicate adverse effects associated with HPV vaccination. Finally, our evaluation suggests that an expanded use of HPV vaccine into more diverse populations, particularly those living in low-resource settings, would provide numerous health and social benefits.

## Introduction

Vaccination is the most successful method to control infectious diseases in terms of both cost and effectiveness. Human papillomavirus (HPV) belongs to a large family of more than 170 double-stranded DNA viruses of which approximately 40 mucosal types are commonly transmitted mainly via sexual activity. Two prophylactic HPV vaccines have been approved by the Food and Drug Administration (FDA) in the USA: the bivalent Cervarix^¯^ (GlaxoSmithKline, Middlesex, UK) for prevention of infection with HPV types 16 and 18 and the quadrivalent Gardasil^¯^ (Merck Sharp & Dohme, USA) for HPV types 6, 11, 16, and 18. Both HPV vaccines can protect females against cervical pre-cancers (CIN).

Several studies have demonstrated that both the bivalent and quadrivalent HPV vaccines are safe (1 2 3). Each has shown long-term durability for protection against primary infections caused by the types of HPV viruses targeted by the respective vaccines along with a moderate degree of cross-protection against some non-targeted HPV viruses, most notably HPV 31, 33, and 45 (4). However, there are several ongoing controversies surrounding compliance with the vaccination recommendation, which at times has involved government health agencies.

It is important to emphasize that the HPV vaccines are not a therapeutic treatment for any HPV-associated disease that might exist at the time of vaccination, nor will it invariably protect against diseases that are caused by types of HPV not targeted by the vaccines. Furthermore, HPV vaccines are not recommended for females <9 years old or individuals that are pregnant. Lastly, Cervarix^¯^ (GlaxoSmithKline) is not licensed for use in males at this time.

Despite the efforts by public health agencies in the United States, the coverage of HPV vaccination remains low. Among adolescent females and males aged 13-16 years, only 33.4 and 6.8%, respectively, had received the three recommended doses of the HPV vaccine in 2012 (5). In June of 2013, the Japanese Ministry of Health partially suspended its HPV vaccination program due to several reported adverse events following HPV immunization (6), which demonstrates that immunization programs can be seriously compromised by safety and possible political concerns. However, much of the published scientific data regarding the safety of HPV vaccines could be influenced by conflicts of interest such as receiving advisory board fees and grant support with commercial interests from the competitive HPV vaccine market. Therefore this review aims to examine, independently of the competing vaccine manufacturers, the current evidence from the peer-reviewed scientific literature referring to the potential adverse effects associated with HPV vaccination.

## Adverse events

One systematic review that involved a total of 29,540 individuals showed that the most frequently reported adverse event related to the HPV vaccines was pain and swelling at the injection site followed by fatigue, fever, gastrointestinal symptoms and headaches (7).

In Japan, HPV vaccination was recommended by the government in April 2013. However, several adverse effects such as complex regional pain syndrome were reported by the Japanese media, which led to a suspension of both the bivalent and quadrivalent HPV vaccines by the Japanese government two months later, in June 2013. Together with the government decision, the media reports also created distrust in the Japanese public that led to a further decrease in HPV vaccination coverage.

Ueda et al. (8) reported that in Japan, between 2012 and 2014, the rate of vaccination against HPV in girls from the 7th grade had plunged from 65.4 to 3.9% and it also decreased significantly for girls in the 8th-10th grades. Another publication from Japan clinically analyzed 44 girls between the ages of 11 and 17 years that complained of several adverse events following HPV vaccination with either the bivalent or the quadrivalent vaccine. Among them, 4 were excluded due to a diagnosis of another disease. Of the remaining 40, the main clinical manifestations reported in the study were: headaches (70%), general fatigue (53%), coldness of the legs (53%), limb pain (50%), limb weakness (48%), difficulty in getting up (48%), orthostatic fainting (43%), decreased ability to learn (43%), arthralgia (43%), limb tremors (40%), gait disturbances (40%), disturbed menstruation (35%) and dizziness (30%). Moreover, a high incidence of chronic limb pain was reported, usually complicated by violent, tremulous involuntary movements. After clinical evaluation, the authors concluded that the observed symptoms could be best explained by an abnormal peripheral sympathetic response (9).

A group of four clinicians clinically examined 3 young women who were diagnosed with secondary amenorrhea to analyze a possible association between primary ovarian failure and HPV vaccination. Their serological data showed increased follicular stimulant hormone and luteinizing hormone. In addition, auto-antibodies specific to the ovaries and the thyroid were detected, which the authors suggest might have been triggered by the HPV vaccine (10). Furthermore, the authors claimed that the Safety Review Committee had failed to consider these autoimmune manifestations, which although non-specific and thus not fitting a well-defined autoimmune condition are still severely disabling.

In disagreement with this publication, Pellegrino et al. (11) wrote a letter to the editor arguing that premature ovarian failure would not necessarily be related to HPV vaccinations. The authors retrieved all cases with a diagnosis of premature ovarian failure or other ovarian failures from the Vaccine Adverse Event Reporting System database and from the United States National Inpatient Sample database. No increase was seen in the number of girls aged 11-17 years who had been diagnosed with ovarian dysfunction following HPV vaccination compared to non-vaccinated girls.

As an opposing response to the previous letter, Colafrancesco et al. (12) suggested that the absence of premature ovarian failure on the list of possible adverse reactions in the HPV vaccine product leaflet could lead to an underreporting of this potentially vaccine-associated effect. It was additionally argued that the analyzed databases cannot be used to establish the presence or absence of causality.

A particularly concerning report showed post-mortem evidence that viral components contained in the HPV vaccine Gardasil^¯^ were capable of crossing the blood-brain barrier, which was suggested to trigger cerebral vasculitis, a severe form of blood vessel inflammation in the brain that can lead to severe autoimmune disorders and even death (13). While the authors presented two cases of young women who died within months after or during the vaccination protocol for Gardisil^¯^, the source of the HPV capsid proteins detected in brain blood vessels by their immunocytochemistry could not be directly attributed to the vaccine. The same authors wrote a letter to the editor concerning anti-vaccination activism versus anti-vaccination based on science (14).

Suba et al. (15) raised many questions in a letter to the editor regarding the effect of HPV vaccine in cervical cancer incidence. Since 2006, the FDA has been concerned with the potential for Gardasil^¯^ to enhance disease among a subgroup of subjects who had shown persistent infection with the HPV types targeted in the vaccine at baseline (16). Another concern is that HPV vaccinations could actually increase the global incidence of cervical cancer-related mortality by reducing detection due to reduced surveillance and screening (16).

An association between HPV vaccination and autoimmune manifestations comparable to systemic lupus erythematosus (SLE) has also been investigated (17). The authors analyzed six women with family and/or a personal history of autoimmune-rheumatic conditions. In all cases, a definitive immunosuppressive response was observed suggesting that individuals with SLE manifestations after HPV vaccination may be limited to women with these characteristics. Additionally, this publication suggests that further studies are required to assess the safety of HPV immunization in patients with autoimmune-rheumatic diseases or those at risk of autoimmunity. It further identified the potential beneficial effects of preventive immunosuppressants. Similarly, a recent report emphasized that potential risks must be carefully considered and evaluated, mainly in individuals who may develop autoimmune diseases either because of their genetic background or prior history of adverse reactions to vaccinations (18).

A possible bias that could influence all clinical trials that have evaluated the safety of both Gardasil^¯^ and Cervarix^¯^ is that placebo groups were often given injections that included the active aluminum adjuvant. Safety concerns exist regarding the aluminum, which is widely used as a vaccine adjuvant. Despite its strong neurotoxic potential, the bioaccumulation of aluminum in the brain appears to occur at a very low rate in normal conditions. Recently, mouse experiments designed to assess the biodistribution of vaccine-derived aluminum have demonstrated that continuously increasing doses of the poorly biodegradable adjuvant may become insidiously unsafe, particularly in cases of repetitive, closely-spaced vaccinations that may alter the blood-brain barrier (19). Other animal models have shown that injected nano-aluminium adjuvant particles can travel from the injection site to distant organs such as spleen and brain (19). Also, other previous studies confirmed the triggering of deleterious immune-inflammatory responses in neural tissues (20,21).

In 2011, the Vaccine Adverse Event Reporting System committee confirmed a strong temporal relationship between the HPV vaccine administration and the onset of anaphylactic reactions. This causality conclusion was based on 36 cases that presented temporality and clinical symptoms consistent with anaphylaxis (22). Additionally, a recent publication reported symptoms of orthostatic intolerance (tachycardia syndrome) and other symptoms consistent with autonomic dysfunction in a population that received the quadrivalent HPV vaccination (23). The authors analyzed 35 young woman who reported adverse symptoms after receiving the HPV vaccination, such as orthostatic intolerance (in all patients), nausea, chronic headache, fatigue, palpitations, reduced cognitive function, skin changes, intermittent tremor/myoclonic twitches, neuropathic pain, sleep disturbances, and muscular weakness in more than half of the patients at the time of the examination. The symptoms were reported to appear in 24% after the first vaccination, 51% after the second and 25% after the third vaccination.

Recently, Dr. Martínez-Lavín reported that some potentially preexisting illnesses are often difficult to diagnose and may have overlapping clinical features such as a dysfunction in the sympathetic nervous system, small fiber neuropathy and fibromyalgia. The article suggests that small fiber neuropathy and dysautonomia could be a common underlying pathogenesis in the group of rare, but severely reacting individuals after HPV vaccination. The author emphasized that clinicians should be aware of the possible association between HPV vaccination and the exacerbation of these difficult to diagnose and painful dysautonomic syndromes (24).

## An overview of HPV vaccination in Brazil

In June of 2006, the National Health Surveillance Agency (ANVISA) approved both prophylactic HPV vaccines for use in Brazil. However, vaccinations were only made available in private clinics since the Brazilian Ministry of Health had not concluded an evaluation for their incorporation into the Public Health system. Starting in March of 2014, the HPV quadrivalent (Gardasil^¯^) vaccine was included in the national immunization program for girls aged 11-13 years old. The vaccine schedule adopted by the Brazilian Ministry of Health involves three doses at 0, 6 and 60 months. The city of Campos dos Goytacazes in the state of Rio de Janeiro has included the quadrivalent vaccine against HPV in the municipal vaccination program using its own funds since September 2010 for residents between the ages of 11 and 15 years. The National System of Notification (SINAN) of the Brazilian Ministry of Health reported a total of 430 local and systemic events in 36% of the persons who received vaccinations in this program that were stratified by dose received in the three dosage administration protocol. No serious adverse events or hospitalization were reported and there has been a 55% reduction in the incidence of genital warts observed in women under 21 years old (25).

Assuming a coverage >90% of pre-teen girls, the implementation of the HPV vaccine in the Brazilian Amazon region is expected to reduce the incidence of cervical cancer and associated mortality from this disease by 42%. This region has the highest mortality rate from cervical cancer in the country (26).

A recent study (financed by Merck & Co., Inc. NJ, USA) evaluated a school-based HPV vaccination program in the City of Barretos, State of São Paulo, where the vaccine coverage rates were high and similar between public and private schools. However, according to the authors, the results achieved from this study may not be extrapolated for other regions in Brazil, mainly for those with limited access to schools in the rural and country areas (27).

## Recommendations and conclusions

Considering that HPV vaccines, like all other vaccines, may not protect all vaccinated individuals, regular cancer screening programs should be maintained irrespective to whether or not a person receives HPV vaccination. It remains to be determined if the newer HPV vaccines against up to seven specific HPV genotypes associated with cancer increase the efficacy of preventing the onset of CIN and cervical cancer, which is already high with current vaccine formulations. However, it has been hypothesized that an increase in the prevalence of other HPV types may occur due to a reduced competition during natural infection, although recent studies found no increase of non-vaccine HPV types, which would be suggestive of type-replacement (28,29).

Duration of efficacy is a key question when discussing the HPV vaccines. According to Dr. Harper, if the duration is at least 15 years, then vaccinating 11-year-old girls will protect them until they are 26 years old, which would prevent some pre-cancers, but primarily postpone most cancers. If duration of efficacy is less than 15 years, then most cancers are not prevented, only postponed. Administration of boosters after the diminishing antibody production elicited by Gardasil^¯^ could extend the duration of efficacy, but would lead to a significant escalation in costs. It would also be a challenge to identify those women in need of revaccination (30). Since the quadrivalent HPV vaccine was approved by the FDA in the USA in June 2006, it will take at least another 15-20 years before the long-term efficacy of these vaccines becomes evident. In December 2014, the FDA approved the so-called HPV9 Gardasil^¯^ vaccine that includes direct protection against HPV types 31, 33, 45, 52 and 58 in addition to the HPV types 6, 11, 16 and 18 of the quadrivalent vaccine.

Although vaccines undoubtedly reduce the incidence of several infectious diseases, which strongly supports that the benefits outweigh the risks, side effects still need to be closely monitored and reported without bias. For HPV vaccines, the body of evidence suggests that their potential to elicit life-threatening side effects is very low with autoimmune responses being the greatest concern. While the reduction in the occurrence of genital warts conferred by the HPV types present in the vaccines strongly suggests an ultimately lower incidence of HPV-positive cancers, the time period since the initial vaccinations has not been sufficient to determine the absolute reduction in cervical cancer, the major benefit expected. For this reason, it is important to stress the need to maintain routine surveillance for lower genital tract diseases especially in women, even if there is widespread use of the HPV vaccine.

We also strongly believe that a regular systematic review of the literature by qualified individuals with no financial interests should be conducted. In many instances, financial resources originate from parties within the competitive HPV vaccine market, which certainly have economic interests, causing major complications in interpreting the results and conclusions from the various studies. Funding also comes from foundations and organizations that are strongly against vaccination programs.
